# Perceptions of a self-guided web-based exercise programme for shoulder pain after spinal cord injury: A qualitative study

**DOI:** 10.1038/s41393-023-00877-3

**Published:** 2023-01-26

**Authors:** Verna Stavric, Nicola L. Saywell, Nicola M. Kayes

**Affiliations:** 1 Rehabilitation Innovation Centre, School of Clinical Sciences, Auckland University of Technology, Auckland, New Zealand; 2 Centre for Person Centred Research, School of Clinical Sciences, Auckland University of Technology, Auckland, New Zealand

**Keywords:** Rehabilitation, Spinal cord diseases

## Abstract

**Study design:**

Qualitative study.

**Objectives:**

The benefits of exercise to reduce shoulder pain in people with spinal cord injury (SCI) are well documented. Digital health interventions offer a potential solution to overcome barriers to access rehabilitation support for exercise. The aim of this project was to gain people’s perspectives to inform the development of a self-guided web-based exercise intervention. Shoulder Pain Intervention delivered over the interNet (SPIN) is a self-guided web-based intervention to prescribe, monitor, and progress evidence-based exercises for people living with SCI and shoulder pain.

**Setting:**

Community in Auckland, New Zealand.

**Methods:**

The Person-Based Approach was used as the framework. Using an Interpretive Descriptive methodology, data were collected in individual and focus group interviews, exploring participants’ perceptions of this intervention idea. Data were analysed using conventional content analysis.

**Results:**

Sixteen participants took part and asked *Is it right for me?*. This had three main sub-themes. *Should I use it?*, whether I believe it will work for me right now; *Can I use it?*, whether I can operate the intervention competently and confidently and *Will I use it?*, whether it will be responsive to my unique needs, and keep me coming back.

**Conclusions:**

Participants expressed their expectations and tipping points when considering using an intervention like this. These findings will inform and guide design and development of an acceptable technology-based intervention to increase the likelihood of engagement with a self-guided web-based exercise programme. The model developed from these themes could be used to inform future self-guided intervention development.

## Introduction

Management of shoulder pain in wheelchair users living with spinal cord impairment (SCI) often includes exercise-based rehabilitation. A programme of stretches and strengthening exercises has been shown to significantly reduce shoulder pain [1–4]. However, many people with SCI (pwSCI) who experience shoulder pain do not engage in these exercises [5]. They cite barriers to exercise and rehabilitation opportunities including limited access to knowledgeable health professionals, poor physical accessibility, and transportation difficulties [6]. Even when these barriers are addressed, persisting with exercises is difficult [5].

Self-guided web-based interventions could address these barriers and have been implemented successfully in other populations experiencing persistent pain [7]. However, existing web-based interventions [8–10] for pwSCI require ongoing input and monitoring from a clinician or do not provide structured exercise progression for shoulder pain.

Shoulder Pain Intervention delivered over the interNet (SPIN) is planned to be a self-guided web-based intervention using decision tree algorithms [11] to prescribe, monitor, and progress evidence-based exercises for pwSCI with shoulder pain [12]. The development of SPIN is supported by the Person-Based Approach (PBA) [12, 13]. The PBA approach seeks a deep understanding of the perspectives and psychosocial context of potential users through employing iterative qualitative research [13] and incorporating existing theories. Keeping users’ needs and contexts in focus maximises engagement and effectiveness of an intervention [14, 15]. As such, understanding the psychosocial context of future users of SPIN is a critical component in this intervention development process. To that end, this study explored the experiences and perspectives of pwSCI regarding the possibility of a self-guided web-based exercise programme to inform the development of SPIN.

## Methods

### Study design

This Interpretive Descriptive study [16] explored impressions and opinions of the possibility of a self-guided web-based exercise programme to treat shoulder pain. The project was approved by the Auckland University of Technology Ethics Committee (AUTEC) 18/263. A stakeholder group, formed as part of the larger project, was consulted to gauge how findings resonated with personal insights and experiences.

### Participants

People living with SCI were eligible for inclusion if they resided in New Zealand; were not engaged in a rehabilitation programme; were over 16 years old; had the capacity to give informed consent; were predominantly wheelchair users; and had experienced shoulder pain within the past 2 years. Participants were excluded if they were unable to communicate with the researcher for data collection.

### Sampling and recruitment

Purposeful sampling was used for diversity in demography and to capture a breadth of perspectives and experiences. Advertisements were distributed within SCI community and rehabilitation providers (e.g. Spinal Support NZ, Neuro Rehab Results). Information was also circulated through social media sites and professional and personal networks. Our sample size was primarily determined by our goal to capture a breadth and diversity of experiences and we aimed to continue sampling until sufficient diversity was reached. We also drew on the concept of information power as a final check on sample sufficiency [17].

### Data collection

Consent was obtained and recorded before data collection began. We collected data using individual and group semi-structured interviews (see Table 1 for interview guide) at participants’ preferred location. We used probes to explore concepts of usability, safety, motivation, action planning, and progression. Participants were also asked about helpful features from websites or apps they currently used. We took notes and interviews were audio recorded, then transcribed verbatim.

**Table 1:** Interview guide.

**Part 1 Sample of progressive interview questions**
“What is your initial thought when you think of using a web-based exercise programme to treat your shoulder pain?”
“Now what if it is intended that you use this programme entirely independently?”
“What if the exercise programme will be set up and progressed without you seeing or being in contact with a health professional?”
**Part 2 Probes and images of current web-based or app-based programme features are presented for feedback and comment**
“What do you think of these features when thinking of a web-based exercise programme?”
“How would these features help with any of the concerns you raised earlier?”

### Data analysis

We analysed the data using conventional content analysis [18], reading transcripts in conjunction with the field notes. VS inductively coded the data, using exact words from the text, to capture key concepts and maximise descriptive and interpretive validity [19]. A coding framework was derived from initial coding of the majority of transcripts (*n* = 9). VS exported all transcripts and the coding framework into NVivo 12 [20] and analysed the remaining transcripts, identifying meaningful clusters. All authors met regularly to discuss and contribute to theme construction. Preliminary themes were presented to the stakeholder group to check for resonance [21], and inform final refinements.

## Findings

### Participant demographics

Sixteen pwSCI with shoulder pain took part in individual (*n* = 8) and focus group (*n* = 8, 4 groups) interviews (Table 2). In one focus group, a support worker also contributed. Participants’ ages ranged from 30 to 67 years. We achieved diversity on the majority of key characteristics as reflected in Table 2, and the final sample has good information power in the context of the study aims, sample specificity, quality of dialogue, and analytical strategy.

**Table 2:** Participant demographics.

Demographic item	Category	Number of participants
Age (years)	30–44	4
	45–59	9
	60+	3
Ethnicity	NZ European	12
	Māori^a^	1
	Pasifika	3
Level of injury	Tetraplegia	7
	Paraplegia	9
Completeness of injury	Complete	11
	Incomplete	5
Time since injury (years)	0–10	2
	11–20	5
	20+	9
Living setting	Urban	12
	Rural	4
Wheelchair type	Manual	15
	Power	1
Interview type	Focus Group	8
	Individual Interview	8

^a^Indigenous population of New Zealand.

### Main findings

Participants engaged in an evaluative process when considering the programme, asking *Is it right for me?*. Our findings suggest this was a nuanced and multi-layered process that consisted of supporting questions or themes to help inform the overarching question. These were: *Should I use it?*, *Can I use it?* and *Will I use it?*.

**Fig. 1:**
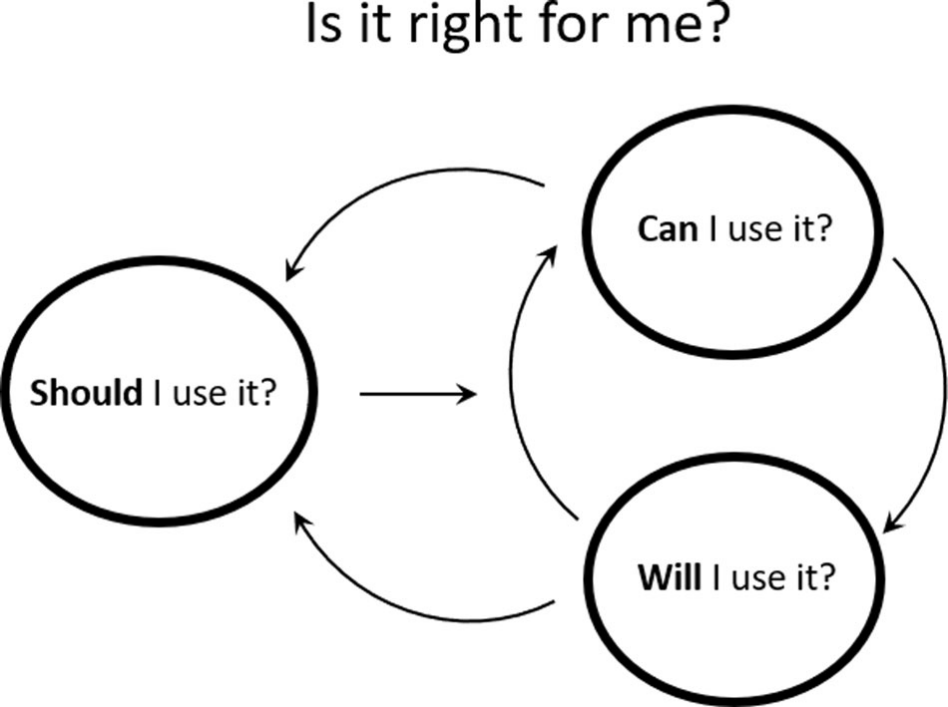
Evaluative process when considering a self-guided web-based programme. Overview of key themes highlighting the evaluative process people with SCI work through when thinking about the possibility of a self-guided web-based exercise programme.

The relationship between these themes is presented in Fig. 1. *Should I use it?* reflects the first step in deciding to take up the programme. If participants determine that the programme has fit for them, they ask *Can I use it?* and *Will I use it?*. If their evaluation suggests that the programme is not responsive to their needs, they may cycle back to questioning *Should I use it?*. This in turn may make them reconsider *Is it right for me?*. The model reflects this cyclical process in deciding if the programme is right for them.

### Theme 1: Should I use it?

*Should I use it?* represents the first step, deciding whether to start using the programme. Participants identified several factors that were important in their initial appraisal.

#### Credibility

Participants required their first impression to be of a credible programme which will meet their needs. Having the programme recommended by a trusted source was preferable to randomly finding it and having to evaluate it independently. Participants identified professional endorsement to be of value, but highlighted that connection with and learning from peers with lived experience was even more powerful.

[…]if you’ve been set up for it by someone you know and trust, either the spinal unit or the physio you’ve been working with for a while, that would be different but […] if it’s just something you stumbled across because you Googled for it […] somehow it wouldn’t carry the same weight, or have the same authority. (Jeremy, C6 tetraplegic)

#### Safety

Safety of the programme was important. Clarity on how each user would be screened, without human oversight, helped to determine safety and engender trust. Despite an inherent assumption that exercise programmes delivered and monitored by humans would be safer and be able to be tailored to the unique needs of users, it was acknowledged that this was not always the case.

Mind you, you can get that with human factor anyway. You might diagnose me wrong too so who cares. And one physio might recommend something different to another, doctors are exactly the same. (Tony, C6 tetraplegic)

#### Perceived value

Perceived value included the benefits of exercise generally, the extent to which exercise could address shoulder pain, and whether the proposed programme would address outcomes of value. Participants acknowledged that reduced access to other traditional or preferred treatments would increase the appeal and value of this programme.

I have shoulder pain […] eventually it goes to the […case manager who…] makes a referral to the physio. Then there’s a stand down period of time. Meanwhile I’ve just got to figure out my own kind of remedy. […] probably we’re looking at maybe 4–6 months […] there’s a loss of that whole period of time where I could have got some help. (Eleanor T12/L1 paraplegic)

Past experience with exercise shaped how the programme was perceived, influencing the value placed on exercise and a person’s intention to exercise. While participants were overwhelmingly positive about the value of exercise for addressing shoulder pain, in some situations exercising was not always considered as important or achievable. There was a range of factors, including timing and competing demands, believed to influence uptake of an exercise programme.

As you get older […] looking after your health is the most important thing. When you’re younger […] it’s on the list but it’s not as high up the list. (Michael, T12/L1 paraplegic)

### Theme 2: Can I use it?

Participants identified factors that would influence their interaction and ongoing engagement with the programme. *Can I use it?* captures the notion of using the programme and performing the exercises with confidence.

#### Feeling competent

Participants expected the programme to guide them to perform and progress the exercises correctly, for best effect. Support workers also wanted guidance from the programme to assist pwSCI to exercise accurately and effectively. Participants related concerns they experience in their daily life with respect to provision of support, including challenges with consistency, and confidence in how their care is delivered, which had the potential to affect programme uptake. This was considered particularly pertinent in the context of high support worker turnover or when therapy is provided by less experienced clinicians.

You know when something didn’t quite go right, they (support workers) didn’t have the training and the knowledge to know which way to jump to sort it out, so we’d give up on it you know. (Jeremy, C6 tetraplegic)

Technological competence and usability of the programme also influenced participants’ answer to *Can I use it?*. Recurrent techincal issues were factors that were believed to influence ongoing engagement with the programme. The usability and clarity of the programme must maximise the chance of successful use and effective exercise performance by users of all levels, with minimal support.

I think that something you’d have to consider, is it’s a lot easier not to ask for help and not do the exercise… Yeah just give up. (Robert, C5 tetraplegic)

#### Having control

Participants recognised the autonomy this programme could bring. They endorsed programme features which put them in charge, allowing them to personalise the programme and meet individual needs and preferences. Having agency to make decisions best suited to each individual, could reinforce engagement through a sense of ownership and control.

Being able to tailor your own solutions, I think is a very important aspect. I hate the term that gets bandied around a lot, empowering you to do this thing or the other, but I mean that is precisely what that’s doing, and I believe it’s important. (Jeremy, C6 tetraplegic)

Practical considerations were important, like procuring and securing equipment, and the support they would need to carry out the exercises. Participants who relied on support from others expressed that the helper’s capacity and beliefs influenced exercise completion, positively and negatively. The need to preserve the relationship has the potential to outweigh the drive to request help with an exercise routine, as it can become an additional demand to the usual list of cares.

… and then, the exercises rely on having the TheraBand set up in such a way that you can do the exercises, now somebody has to set me up with that TheraBand, and that person has to be a willing participant. (Robert, C5 tetraplegic)

### Theme 3: Will I use it?

*Will I use it?* reflects participants’ views on the importance of being engaged by the programme to encourage them to keep coming back and complete the exercises.

#### Fostering positive experiences

Participants discussed how positive emotional experiences would be an important hook to encourage users and keep them interested. Participants suggested that early success and progression of exercise performance was one mechanism to achieve this. Being able to track performance and progress was seen as feedback that would encourage users.

The use of messages that give feedback on performance was also considered important. The tone, language and timing of this information were discussed. Some participants felt they would respond to a supportive tone whereas others felt a challenging and competitive tone was more motivating.

You could have of fun with it, you know, and to engage and use it, they could customise it. So, I might have mine that says, ‘you know, come on, you haven’t done it for two days, ‘the ** you doing?!’ And it’s kind of their own reminder so it’s part of the whole orientation. Do you want to remind yourself? I do. (George, C6 tetraplegic)

The use of rewards to encourage return to the programme and focus on the goal was also discussed. Several participants suggested using gamification principles like awarding points for tasks to provide a positive experience and encourage regular use.

It would give me my points for doing the exercises regularly, 3 days a week, 3 times a week, […], if I miss a day then I drop a few points, so that there’s a desire to not miss a week you know, and I’m beating that guy so I’m doing better than he is […] The gaming aspect is to try and engage most people’s inherent competitive nature, even if they’re only competing against themselves. (Lawrence, T12/L1 paraplegic)

#### Seeing progress

Seeing progress in exercise performance or function, was important in promoting programme value. Participants spoke of their sense of success when they realised they could perform more exercises than they thought. This gave them the confidence to do more exercise and engage more in everyday activities. There was an understanding that progress can vary and the goal may be to increase daily activity or function, while keeping pain under control, rather than eliminate all pain. Ensuring meaningful progress is captured was considered formative to providing a personalised programme that accounts for user’s needs and preferences. This was important to ensure ongoing engagement, when there is no clinician involvement.

Yeah, I think the feedback that I would want to see, is a graph of my progress, so that I can see that I’m actually improving. (Robert, C5 tetraplegic)

#### Feeling connected

Participants spoke about how feeling connected helped them stay engaged and feel supported. While they felt this should come from their health professional, they valued support and encouragement from anybody who was interested and invested in their success. This support helped them stay on track and be accountable.

I know from my previous life experience, that I always work better when I’ve got somebody to encourage me, or somebody that’s interested, because they’re interested in how I’m getting on. They don’t want to see me suffering from sore shoulders. Yeah, so that connection might be really important. (Robert, C5 tetraplegic)

If accountability could be built into the system, participants were challenged to consider whether it mattered where it came from. Connection and accountability could come from nominated people, other users, and from the programme despite there not being a health professional involved. However, this was not universally endorsed.

…if it was totally autonomous and there was no personal interaction whatsoever, they may not have all that interest in it, or they lose interest too quickly or whatever, it’s just a soulless computer programme, nobody at the other end. (Robert, C5 tetraplegic)

## Discussion

We used the PBA to explore perceptions, of pwSCI, when considering using a self-guided web-based exercise programme to treat shoulder pain. The overarching question participants asked was *Is it right for me?*. Reinforcing this was a continuous process of revisiting the supporting questions of *Should I use it?, Can I use it?*, and *Will I use it?*.

*Should I use it?* highlighted the importance of credibility and safety when using a web-based intervention. PwSCI accessing web-based physical activity information have expressed a preference for knowing material came from a reliable source [22]. Features that promote credibility and guidance have been shown to increase trust in an intervention in other populations [23].

*Can I use it?* reflected participants’ views about using the programme competently, confidently, and autonomously. The ability to tailor features, including prompts, has been employed by other self-management interventions after SCI [22, 24], increasing the programme’s personal relevance. Our findings confirm those of Singh et al. [9] who reported that a self-management app for pwSCI would need to be intuitive and offer user flexibility and control.

*Will I use it?* related to ongoing engagement and motivation to keep returning to the programme and exercises. Strategies that were identified were consistent with findings from previous studies: being able to monitor progress to measure improvement and stay accountable [15, 22], and using peer connection [22, 24]. Perski et al. found that features which facilitated social support [24] positively influenced engagement [25].

In addition to the identified themes, three concepts were interwoven throughout the data: needing to feel connected, feel competent and have autonomy. These concepts align well with Self-Determination Theory (SDT), which proposes that three basic psychological needs have to be met to be self-determining and autonomously motivated; a need for competence, relatedness, and autonomy [26]. In the context of SPIN, users would need to feel competent using it, feel connected to it or others while using it, and have a sense of control or choice when using it. Development of SPIN will incorporate these concepts into its design.

### SPIN development and roll-out implications

The design goal of SPIN is to incorporate features that can create experiences without a clinician being present. This requires intentional use of intervention features that engender confidence that SPIN is a safe, effective, and personalised programme. This study’s findings confirm previous findings that interventions need to be tailored, theory-based, multimodal, and incorporate behavioural strategies [15, 27]. Drawing on the PBA, our next step is to use what we have learned in this research, and from existing theory and evidence, to develop a set of guiding principles which articulate the intervention design objectives and the features needed to achieve these objectives [12]. Using the PBA in pwSCI with shoulder pain, has highlighted what they need from this self-guided exercise intervention. Our findings highlight that when SPIN is developed and ready for implementation, our roll out plan should include liaising with SCI clinicians and services to promote SPIN as an evidenced-based tool to augment current intervention options. Recommendations of SPIN from these credible sources would increase trust and uptake.

## Strengths and limitations

### Strengths

The tiered process of questioning in this study allowed a depth of discussion and analysis in keeping with the PBA. The range of participants, coupled with the stakeholder group, ensured that findings have transferability to pwSCI. The robust methodological and analytic approach ensured that our findings went beyond a semantic level, to identify a deeper decision-making process, explaining a clinical phenomenon. This model could be applied when reflecting on the development of any self-guided intervention.

### Limitations

Our findings may not be representative of the entire SCI community who live with shoulder pain. The perspectives of Māori, or indeed other indigenous and culturally diverse populations, and those who live rurally were not fully captured in this research. Future work will purposively work with these sub-groups.

## Conclusions

This study used the PBA to investigate the perceptions and experiences of pwSCI with shoulder pain to inform the development of a self-guided web-based exercise intervention. The involvement of participants in the early development phases has enabled us to identify key decision-making points when considering intervention uptake and ongoing use. This will inform the design of the SPIN programme and will act as a model which could inform the development of other self-guided interventions.

## Supplementary information

Reproducibility checklist

## Data Availability

Data that was generated and analysed during this study can be found within the published article. Additional data are available from the corresponding author on reasonable request.
